# Evaluating the Effects and Safety of Intravenous Lipid Emulsion on Haloperidol-Induced Neurotoxicity in Rabbit

**DOI:** 10.1155/2014/949262

**Published:** 2014-05-29

**Authors:** Mohammad Moshiri, Amir Hooshang Mohammadpour, Maryam Vahabzadeh, Leila Etemad, Bahram Memar, Hossein Hosseinzadeh

**Affiliations:** ^1^Department of Pharmacodynamy and Toxicology, School of Pharmacy, Mashhad University of Medical Sciences, Mashhad, Iran; ^2^Medical Toxicology Research Center, School of Medicine, Mashhad University of Medical Sciences, Mashhad, Iran; ^3^Pharmaceutical Research Center, Mashhad University of Medical Sciences, Mashhad, Iran; ^4^Department of Pathology, Imam Reza Hospital, Mashhad University of Medical Sciences, Mashhad, Iran; ^5^Pharmaceutical Research Center, School of Pharmacy, Mashhad University of Medical Sciences, P.O. Box 91775 1365, Mashhad, Iran

## Abstract

There are many reports on the effect of intravenous lipid emulsion (ILE) as an antidote in drugs related toxicities. We determined the effects of ILE on neurotoxicity of haloperidol (HA), a highly lipophilic antipsychotic, as a model of antipsychotics poisoning. We used six groups of five male rabbits. Two groups received distilled water intravenously followed by infusions of either 18 mL/kg of normal saline or ILE 20%, after 30 minutes. The third group received 18 mL/kg of normal saline after HA (2.6 mg/kg) administration. The three other groups received ILE 20% solution (6, 12, and 18 mL/kg) following HA injection. Catalepsy scores, temperature, pupil size, and mortality rate were measured at 0, 0.5, 1, 2, 3, 4, 8, and 24 hours after HA administration began. Blood and tissue samples were taken from all animals at 24 hours or at death time for biochemical, cell count, and pathological studies. ILE reversed cataleptic scores, miotic pupils, and hypothermia of HA intoxication much faster than normal saline (*P* < 0.001). Biochemical complications and mortality rate of the animals were significantly higher in the HA + 18 mL/Kg ILE group. ILE reversed sings of HA neurotoxicity; however, synergistic effect of high dose of ILE and HA increased complications and mortality.

## 1. Introduction

Intravenous lipid emulsion (ILE) has been known as a novel antidote against lipophilic xenobiotics intoxication in animal studies and some case reports [1–4]. The numbers of animal studies or case reports evaluating antidotal effects of ILE against nervous system toxins are not as much as its cardiac and hemodynamic effects [4, 5].

Acute poisoning with antipsychotic drugs is an important cause of referring intoxicated patients to the hospital [6, 7]. Haloperidol (HA), a classic butyrophenone antipsychotic, has been used as model of antipsychotic intoxication in several studies. Moreover, it has been suggested that some of HA properties, such as high lipid solubility and water-soluble metabolites with low pharmacological activity, make ILE suitable for the treatment of HA intoxication [2]. HA intoxication can induce catalepsy and other neurological manifestations that can be evaluated in animal models [8]. As it is a potent typical antipsychotic, HA intoxication has shown higher incidence of catalepsy and extrapyramidal manifestations. Hypothermia and anticholinergic manifestations are other common signs of HA intoxication. The mainstay of treatment in acute HA overdose is supportive and there is no specific antidote [9].

In this study, we tried to determine whether ILE would have beneficial neurologic effects in the setting of HA toxicity.

## 2. Material and Methods

### 2.1. Animals and Preparations

Thirty male New Zealand rabbits with body weight between 2500 and 3000 g were used. They were housed in individual cages with 12-hour light/dark cycle and easy access to food and water. The experiments were approved by the Ethics Committee for Animal of MUMS (Mashhad University of Medical Science).

Haloperidol ampoules, 5 mg/mL, (Exir Pharmaceutical Co., Iran) were used and each 1 mL of HA, 5 mg, was diluted up to 5 mL with aseptic distilled water (ADW). ILE 20% (Intralipid emulsion, Fresenius Kabi AB, Spain) and aseptic normal saline (NS) were also administered intravenously.

### 2.2. Procedures

#### 2.2.1. Animal Groups and Solutions Administration

Thirty rabbits were divided into six groups as follows.

 (a) Negative control group (NC): they received ADW and then 18 mL/kg of NS.
 (b) ILE 0 group: they received ADW and 18 mL/kg ILE 20%.
 (c) NS group: 18 mL/kg of NS was administered after receiving HA infusion.
 (d, e, and f) These 3 groups received ILE 20% solution with 6, 12, and 18 mL/kg doses (ILE 6, ILE12, and ILE18 groups), respectively, following HA administration.

These doses had been selected based upon previous studies [10–12].

A 24 G venues cannula was inserted into marginal ear vein of rabbits in aseptic conditions. Subsequently, either diluted HA or an equal volume of ADW was infused intravenously within 15 minutes. HA was administrated at a dose of 2.6 mg/kg body weight of rabbits (equal to 2.6 mL/kg of diluted HA solution or ADW). Zero time was considered when HA or ADW infusions began. ILE 20% or NS were intravenously infused, in an infusion rate of 4 mL/min, at 30 min. At the end of the infusion, the cannulas were removed. All studies started at 08:00 am.

#### 2.2.2. Animal Toxicity Tests

Pupils size, catalepsy score, body temperature, and mortality rate of rabbits were evaluated before starting drug or solution administration at baseline (time 0) and through 24 hours at 0.5 hour (just before infusion of ILE 2% or NS), 1, 2, 3, 4, 8, and 24 hours.

The pupils size were assessed in millimeter based on the diameter of right eye pupil, when the rabbits were placed on laboratory table in an identical location and at equal direction under similar brightness of light at all evolution times.

Body temperatures of animals were evaluated at all evolution times by a rectal thermometer according to the method previously reported in the literature [13].

Catalepsy of animals was assessed by Banerjee et al. method with some modification [14]. Three scores of catalepsy were as follows.

- The animal right foreleg was brought round behind its back. The latency of returning the malpositioned forelimb to normal state was measured in seconds. Sixteen seconds was considered as the cutoff time to protect the animal limb against possible injuries.
- The experimenter abducted, internal rotated, and extended the right forelimb of the animal in abnormal position. The latency of returning the limb to its normal position was recorded. The cutoff time was considered 60 seconds.
- The rabbit was placed in a nearly upright position with its forepaws over a step. The latency of the animal being able to maintain this posture was recorded. The cutoff time was 4 min (240 seconds).

At each run, every part of catalepsy scores was measured twice and the means of data were recorded.

#### 2.2.3. Biochemical and Histopathological Studies

Blood samples were drawn from all animals after 24 hours or at the time of death if they died sooner than 24 h. One milliliter of whole blood was drawn into a test tube containing anticoagulant (citrate) and the cells blood count was measured by cell counter (Sysmex KX21n, Japan). Biochemical tests (alanine transaminase (ALT), aspartate transaminase (AST), alkaline phosphates (ALP), blood urea nitrogen (BUN), serum creatinine (Cr), amylase, and lipase) were performed by autoanalyzer (B.T1500 plus, Roma, Italy) on 0.3 milliliter of animals' blood serums.

Autopsy was performed immediately after sacrifice or at death time if they died sooner than 24 h. Tissues from various organs, right hemisphere of the brain, right lung, and right kidney were fixed in 10% buffered formalin and embedded in paraffin. Right hemispheres of brain were divided into four sections: frontal lobe, parietal and basal ganglion region, occipital lobe, and cerebellum and medulla. Fragments of the various organs were sectioned at 70 Wm by vibratome and stained with hematoxylin and eosin. The tissue samples were evaluated by a single pathologist. All tissues and blood samples were coded and all experiments were blindly performed.

Further sections of the brain were processed in TUNEL procedure to detect evidence of apoptotic cells. Sections were stained according to the manufacturer instructions (In Situ Cell Dead Detection Kit, Roche). In brief, sections were dried onto poly-l-lysine-coated slides, treated with proteinase K (20 mg/mL), and incubated with TdT enzyme. Percentages of apoptotic nuclei were calculated by counting them being distributed within 100 nuclei.

### 2.3. Statistical Analysis

Statistical analysis of all variables was performed by SPSS 11.5. Two-way repeated-measures ANOVA was used to evaluate differences in continuous numerical variables of different groups. Tukey *t*-test was performed as post-test if the *P* value was less than 0.05. Kruskal-Wallis ANOVA test were used if necessary. Dunn test was set as posttest only if the result was positive; the *P* value was less than 0.05. Fisher's exact test was used to evaluate differences in mortality rate of different groups. The alpha level was set at 0.05.

## 3. Results

### 3.1. Temperature and Pupil Size

**Figure 1:**
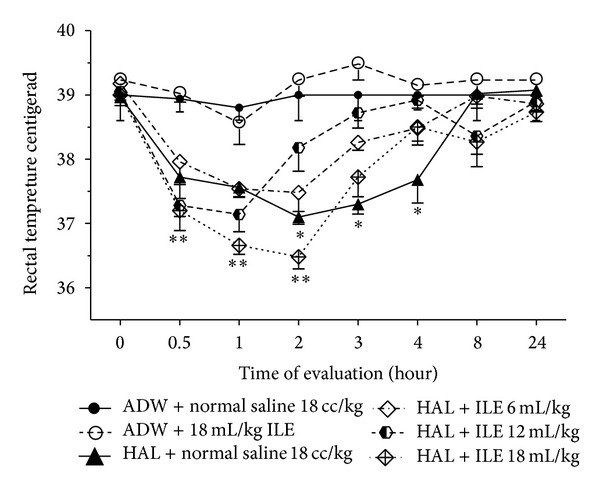
Evaluation of rectal temperatures changes in haloperidol (HA) intoxicated rabbits that received various doses of intravenous lipid emulsion (ILE) or normal saline. Starting of HA or AWD infusion: zero time. ILE or normal saline were infused at 0.5 hour. Difference between ILE 6 and ILE 12 with NC was not significant at second hour. *P < 0.05, **P < 0.01, NS: not significant, ADW: aseptic distilled water.

All HA-treated rabbits suffered from hypothermia (*P* < 0.01) (Figure 1). Rectal temperatures of the animals in ILE 12 and ILE 6 groups returned to normal at the second and the third hours, respectively (Figure 1). The NS group was hypothermic until 4 hours after starting HA injection.

**Figure 2:**
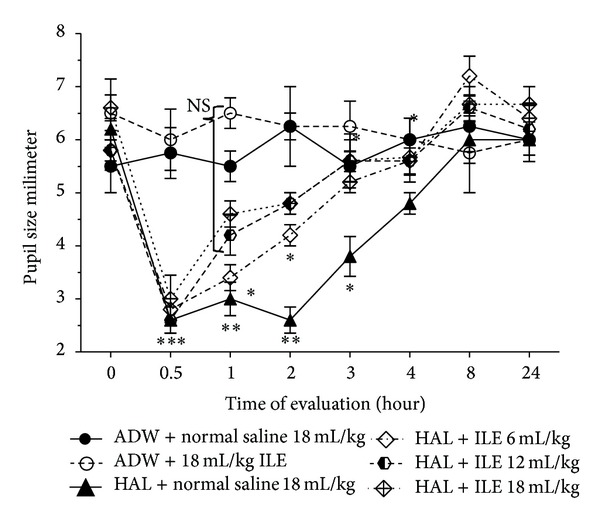
Evaluation of pupil size (millimeter) changes in haloperidol (HA) intoxicated rabbits that received various doses of intravenous lipid emulsion (ILE) or normal saline. Starting of HA or AWD infusion: zero time. Infusion of ILE or normal saline was started at 0.5 hour. *P < 0.05, **P < 0.01, ***P < 0.001, NS: not significant, ADW: aseptic distilled water.

Miosis was presented in all HA-administrated rabbits (*P* < 0.0001) (Figure 2). By the end of ILE 20% administration (1 hour after starting HA injection), pupil miosis in ILE 12 and ILE 18 disappeared; however, NS and ILE 6 were still miotic. The differences between mean pupil sizes of ILE 6 and NS groups with NC group were not significant at the 3rd and 4th hours, respectively (Figure 2).

### 3.2. Catalepsy Scores

#### 3.2.1. Latency of Repositioning of the Foreleg Being Positioned on the Back

**Figure 3:**
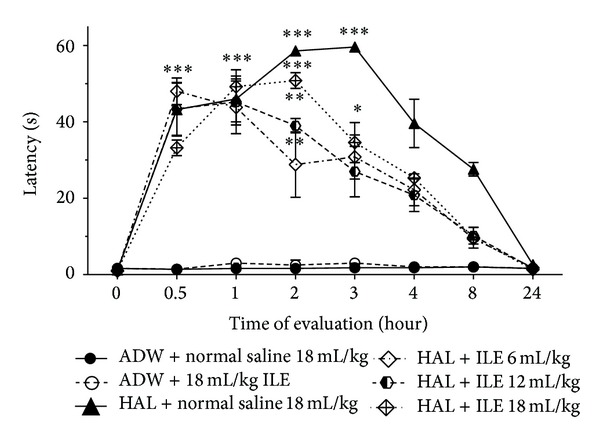
Evaluation of cataleptic score (malpositioned forelimb on back) changes in haloperidol (HAL) intoxicated rabbits that received various doses of intravenous lipid emulsion (ILE) or normal saline. Starting of HAL or AWD infusion: zero time. Infusion of ILE or normal saline was started at 0.5 hour. *P < 0.05, **P < 0.01, ***P < 0.001, ADW: aseptic distilled water. At the second hour, *P*  value of difference between means of latency time of ILE 12 and ILE 18 with HAL + normal saline <0.05. At the third hour, *P* value of difference between means of latency time of ILE 6, ILE 12, and ILE 18 with Hal + normal saline <0.001.

HA-treated rabbits were not able to reposition their forelimbs up to 60 seconds at the first hour of the evaluation. Animals in ILE 6 and ILE 12 groups returned their malpositioned limbs sooner than NS group (*P* < 0.05 and *P* < 0.001, resp.) in the second hour. At the third hour all HA-intoxicated animals which received ILE (6, 12, and 18) had significantly lower cataleptic scores than NS group; however, their mean scores were higher than those in NC group (Figure 3). The results of the 8th hour were similar to the third hour with lesser score. There was no difference between mean catalepsy score of ILE 0 and NC groups at all evaluation times.

#### 3.2.2. Latency of Repositioning the Foreleg Which Was Extended, Rotated, and Abducted

**Figure 4:**
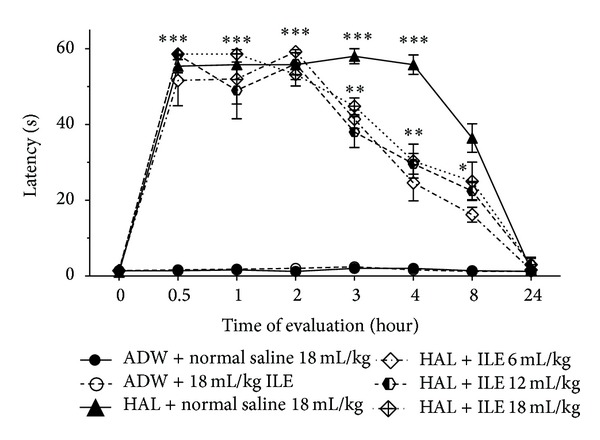
Evaluation of cataleptic score (extended and rotated forelimb) changes in haloperidol (HAL) intoxicated rabbits that received various doses of intravenous lipid emulsion (ILE) or normal saline. Starting of HAL or AWD infusion: zero time. Infusion of ILE or normal saline was started at 0.5 hour.  *P < 0.05, **P < 0.01, ***P < 0.001, NS: not significant, ADW: aseptic distilled water.

The mean time for repositioning the extended foreleg in HA-intoxicated animals was longer than the NC group (*P* < 0.0001) (Figure 4).

The mean cataleptic scores of HA + ILE treated animals (ILE 6, ILE 12, and ILE 18) were reduced at 3rd hour in comparison with NS group (*P* < 0.0001), but they did not reach NC group score (*P* < 0.0001). There was no significant difference between mean catalepsy score of NS group in second and third hours (55.28 ± 2.57 and 55.0 ± 2.0 seconds). All HA-treated animals were cataleptic at 8th hour; however, the means of scores of ILE groups were significantly lower than the NS score (*P* = 0.0022).

#### 3.2.3. Standing Scores of Animals

**Figure 5:**
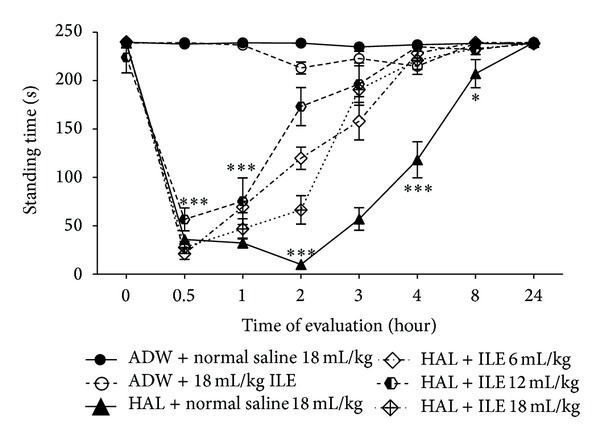
Evaluation of cataleptic score (standing time) in haloperidol (HAL) intoxicated rabbits that received various doses of intravenous lipid emulsion (ILE) or normal saline. Starting of HAL or AWD infusion: zero time. Infusion of ILE or normal saline was started at 0.5 hour. *P < 0.05, **P < 0.01, ***P < 0.001, NS: not significant, ADW: aseptic distilled water. At the 2nd hour, *P* value of difference between means of standing time of ILE 6, ILE 12, and ILE 18 <0.01. At the 2nd hour, *P* value of difference between means of standing time of ILE 6, ILE 12, and ILE 18 with HAL + normal saline <0.001.

After administration of HA, the standing ability of animals was significantly reduced (*P* < 0.0001) (Figure 5). This lasted for 2 hours. At the second hour, animals which received ILE could stand longer than those treated with normal saline (NS group) (*P* < 0.0001), although, there were significant differences between the mean score of ILE groups (time of standing of ILE 12 > ILE 6 > ILE 18) (*P* < 0.05). At 4th hour, the NS group was cataleptic but none of the ILE groups were.

### 3.3. Mortality

All animals were alive at 24th hour (alive = 100%) except 2 rabbits of ILE 18 groups (alive = 60%) (*P* = 0.023, Fisher's exact test).

### 3.4. Laboratory Tests

The mean Cr of NS and ILE 18 groups was increased; however, they were not statistically significant (Table 1). On the other hand, the mean BUN of NS and ILE 18 groups was higher than ILE 0 and NC groups (*P* < 00.1 and *P* < 0.05, resp.). There was no statistically significant difference between means of BUN and Cr in ILE 6 and ILE 12 and control groups (Table 1).

The mean of amylase and lipase of serum was increased in ILE 18 group in comparison with NC group; but it was not significant (Table 1).

HA injection could raise the mean of ALP in NS group in comparison with NC (*P* = 0.0286). In addition, the means of ALP in ILE 6 and ILE 18 groups were raised, but the means of ALP of NC, ILE 0, and ILE 12 groups were the same (*P* values of comparing means of ALP of ILE 6, ILE 12, and ILE 18 with NC groups were *P* = 0.0122, *P* = 0.0728, and *P* = 0.0335, resp.).

The serum AST of ILE 18 group rose significantly (*P* < 0.05). The mean level of serum AST in NS group was also increased but was not statistically significant compared to NC group (Table 1).

The means of serums ALT in ILE 12 and ILE 18 were significantly higher than those in NC and ILE 0 groups (*P* < 0.05). However, they were not statistically higher than mean serums ALT in NS group (Table 1).

We could not detect a significant difference between hemoglobin, hematocrit, red blood cells count, and their indexes in different groups.

**Figure 6:**
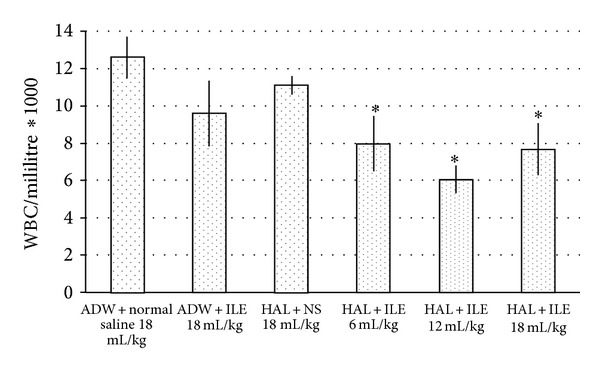
Means of white blood cell (WBC) count in haloperidol (HAL) intoxicated rabbits that received various doses of intravenous lipid emulsion (ILE) or normal saline, at 24 hours after starting HAL or AWD infusion. *P < 0.05, ADW: aseptic distilled water.

There were no statistically significant differences between means of white blood cell (WBC) count in NC, ILE 0, and NS groups; however, means of WBC count in ILE 6, ILE 12, and ILE18 were significantly reduced in comparison with NC group (*P* = 0.0322, *P* = 0.0222, and *P* = 0.0368, resp.) (Figure 6). There were no statistically significant differences of WBC between ILE 6, ILE 12, and ILE 18.

### 3.5. Pathology and Tunel

**Figure 7:**
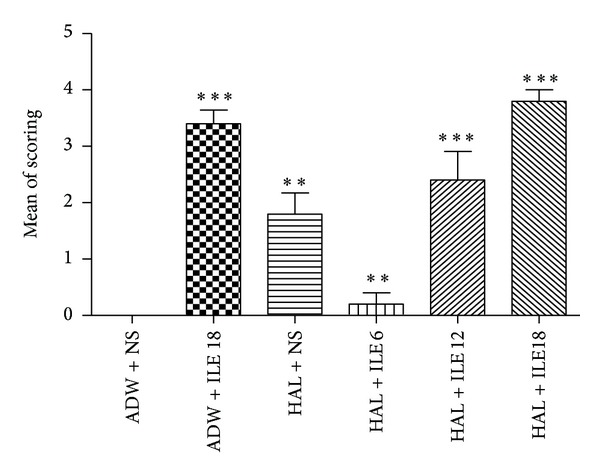
The means of lung lesion scoring of rabbits treated by haloperidol (HAL) and intravenous lipid emulsion (ILE) or normal saline (NS). **P < 0.01, ***P < 0.001, ADW: aseptic distilled water.

**Figure 8:**
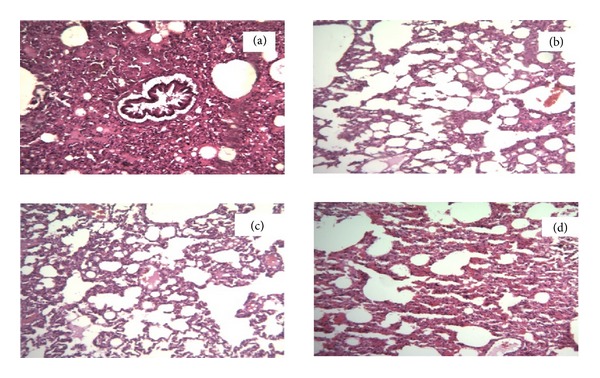
Lung slides of rabbits treated by haloperidol (HAL) and intravenous lipid emulsion (ILE) or normal saline (NS). Magnification = ×400. (a) Received aseptic distilled water and 18 mL/kg ILE 20%. (b) Received HAL and 18 mL/kg normal saline. (c) Received HAL and 12 mL/kg of ILE 20%. (d) Received HAL and 18 mL/kg of ILE20%.

There were 2–5% apoptotic cells in different sections of rabbits' brains that were not statistically significant. The renal pathology of one rabbit in NS group showed acute tubular necrosis pattern. The others had no similar findings. The prevalence of acute tubular necrosis was not statistically significant between different groups. Pathological evaluation revealed inflammatory cell infiltration through the lung tissue of rabbits, especially in ILE 0 and ILE 18 groups (Figures 7 and 8).

## 4. Discussion

There are some reports about the effect of ILE on xenobiotics-induced central nervous system (CNS) toxicity [15]. For instance, ILE could reverse local anesthetic CNS toxicity [16] and it also raised the Glasgow coma scale of intoxicated patients [17, 18]. This study has shown the effectiveness of ILE on CNS manifestations of HA toxicity.

HA is a typical potent neuroleptic that is an antagonist of D2 receptors [19]. It induces catalepsy in a dose dependent manner [20]. The main mechanism of HA-induced catalepsy is mediated by D2 receptor blockade. HA is a lipophilic drug that has octanol/water partition coefficient of 4.3 and volume of distribution of approximately 20 [21]. The most important hypothesis that explains the mechanism of antidotal effect of ILE against CNS intoxication of xenobiotics is lipid sink theory. It suggests that lipophilic drugs or xenobiotics, which induce intoxication, are redistributed from their site of action to a new inert compartment made by ILE in the vessels [22, 23].

It seems that there are some other explanations for antidotal effect of ILE against HA-induced catalepsy. There are some reports that coadministration of voltage calcium channel blockers with HA could increase the catalepsy score of rats and decrease the onset of catalepsy by reducing the neurotransmitter dopamine in striatal region [8, 24]. However, calcium channel blockers cannot induce catalepsy when administrated alone. It is known that flunarizine and cinnarizine, two piperazine calcium channel blockers, can provoke parkinsonism, tardive dyskinesia, and akathisia [25]. It has also been reported that the duration of catalepsy would be prolonged, if the levels of calcium (Ca) and magnesium (Mg) in drinking water were low [26]. On the other hand, ILE and high chain free fatty acid are capable of increasing intracellular calcium concentrations in neurons [27, 28] and also in ventricular muscles cells [29]. Thus, it is suggested that ILE not only reduces HA concentration in target organs based on the lipid sink theory, but also restores the HA-induced catalepsy through increasing the intraneuronal calcium concentration.

Intravenous administration of HA-induced hypothermia in all rabbits and ILE 20% restored the reduced temperature to normal values sooner than normal saline. Lin has suggested that hypothermia-induced HA may be due to a decrease in metabolic heat production as well as an increase in ear blood flow [30]. Boschi et al. have shown that the hypothermic effect of neuroleptic drugs, such as HA, is mediated by peripheral mechanisms, especially alpha 1 adrenergic receptor, more than central receptors. Phenylephrine, an alpha-adrenergic receptor agonist that does not cross the blood-brain barrier, could reverse neuroleptic hypothermia through peripheral vasoconstriction [31]. Surprisingly, Stepniakowski and colleagues, in two separate research projects, have shown rising vascular alpha-adrenergic response to exogenous and endogenous alpha agonist after ILE infusion [32, 33]. It has been also reported that ILE could increase temperature in less than 1% of patients who had received it [34]. However, we found no changes in means of body temperature in ILE 0 group rabbits compared to NC group. Thus, it seems that ILE could reverse hypothermia induced by HA via peripheral vasoconstriction mechanism, besides lipid sink phenomena.

HA-induced miosis was reversed by ILE 20% more rapidly than normal saline. The miosis that resulted from HA administration is more related to peripheral effect of HA than central effects. HA-induced miosis is not mediated by blocking alpha-adrenergic receptors of radial muscles or stimulating cholinergic receptors of circular muscles of iris [35]. Thus, ILE 20% not only reversed the central nervous system symptom of intoxication such as catalepsy, but also could reverse peripheral manifestations of HA intoxication.

Rising BUN and Cr in rabbits happened in NS group and one rabbit of this group suffered acute tubular necrosis. Dopamine causes renal vasodilation which is attenuated by haloperidol [36]. Rabbits belonging to ILE 6, ILE 12, and ILE O groups did not have higher BUN or Cr than the control. However, administration of HA plus ILE 18 mL/kg raised the BUN and Cr more than other groups. It might be due to the synergistic effect of HA and ILE 18 mL/kg and deterioration of rabbits condition due to fat overload syndrome.

Minor elevations of serum aminotransferases commonly happen secondary to HA therapy [37], and there are some case reports and animal studies about liver dysfunction induced by HA [38, 39]. Serums ALP of HA-treated rabbits were high. Administration of ILE (6 and 12 mL/kg) could reduce the elevated HA-induced ALP. However, they did not return to normal levels. Serum AST in NS group was nonsignificantly raised, but ILE could reverse this rising in 6 and 12 mL/kg doses. However, 18 mL/kg of ILE significantly increased the transaminase enzyme levels. As fat overload syndrome of ILE is accompanied with liver damage [40], these complications may be due to the synergistic effect of ILE overload syndrome and hepatotoxicity of HA, although, we detected no liver enzyme elevations in ILE 0 group and the highest dose of our study was much less than doses that induce fat overload syndrome. Hiller et al., in a study on evaluation of the safety of high doses ILE, reported elevation of AST in all doses of ILE in rats and showed the liver damage in histological studies in their highest doses; although their ILE administration doses were extremely higher (20–80 mL/kg) than our doses [41]. It has been reported that liver damage and elevated liver enzymes are consequences of injection of higher doses of HA in rats with fatty liver [39]; however, these two conditions, fat overload liver and fatty liver, are not exactly the same.

HA administration has the possibility of drug-induced leucopenia/neutropenia, especially in patients with preexisting low WBC or coadministration of compounds which could induce leucopenia [42, 43]. Administration of ILE 20% can induce leucopenia as a delayed adverse reaction [34, 44]. In our study, neither NS group, which had received HA plus normal saline, nor ILE 0 group, which had received ILE 20% 18 cc/kg without HA, showed significant leucopenia. However, all groups that were treated by HA and ILE had significant leucopenia. It seems that this is the result of synergistic effect of HA and ILE on WBC count.

We found no difference between amylase and lipase of all groups. Although, daily injection of 2 mg/kg of HA into mice has significantly raised the protein content and the volume of the basal pancreatic juice [45]. Infusion of 1 mg of HA into intrapancreatic arterial attenuated the dopamine-induced pancreatic secretion [36]. Dopamine, whose receptors can be blocked by HA, is also a potent inducer of amylase discharge from the guinea-pig submandibular gland [46]. Pancreatitis secondary to HA treatment is very rare and it is less than atypical antipsychotic drugs [47]. On the other hand, Marwick et al. described elevated serum amylase levels in a bupivacaine intoxicated case that was resuscitated by ILE [48], and Hiller et al. reported higher amylase in high dose ILE infused to rats [41]. However, as previously mentioned, the dose of their study was higher than ours. Some authors believe intravenous fat emulsions may rarely cause pancreatitis [49].

Our histopathological examinations showed the dose dependent lung infiltration due to fat emboli. Lung injuries secondary to ILE infusion are complicated. It seems that two important factors are involved in this topic, underling pulmonary condition and lipid load. Some case series revealed lowered blood oxygen content, increased shunting, and pulmonary vasoconstriction after ILE infusion in patients with respiratory failure and/or sepsis [50, 51], and the pulmonary complication of ARDS patients increased by ILE infusion [52, 53]. However, lower oxygenation or pulmonary vascular changes had not been reported in patients with normal lung function [52, 54]. HA can induce bronchospasm and laryngospasm and reduce pulmonary ventilation and hemoconcentration [55]. It has been also reported that HA had induced hemorrhagic alveolitis [56]. Levine et al. reviewed the delayed complications of nine cases which had been rescued by ILE through 2005–2012. ARDS was the most complication in these cases. However, the authors believed that the lung injuries are more related to their illness than the ILE administration [57].

On the other hand, part of this variation is related to the infusion rate and concentration of ILE that result in lipid load [58]. Jacobovitz-Derks and Derks have reported transient pulmonary edema induced by ILE infusion in dogs, secondary to increased pulmonary arterial pressure. They suggested that pulmonary lesions are induced by neutral fat hydrolysis and pulmonary lipase and the direct fatty acid cell toxicity [59]. Inwood and colleagues believed that intralipid-induced lung injury resulted from hypoxia secondary to vasoconstriction and it could be reversed by indomethacin [60]. High dose and fast administration of ILE result in raising concentration of plasma free fatty acid due to a rise in lipoprotein lipase activation [61, 62]. High concentration of free fatty acid induces vasoconstriction due to impaired endothelium dependent vasorelaxation [63]. Fatty acid infusion stimulates increasing concentration of prostaglandins, potent inflammatory mediators [64], and endothelial dysfunction [65]. Lekka et al. also evaluated the effect of administration of medium- and long-chain triglycerides on pulmonary function. Releasing of phospholipase A(2) and platelet-activating factor secondary to lipids infusion activated inflammatory cells in patients with pulmonary problem. These activated cells enhanced the edema formation, inflammation, and surfactant alterations. These findings have not been shown in non-ARDS patients [54].

HA-intoxicated animals, which received 18 mL/kg of ILE, had the highest mortality rate and they also showed worse laboratory tests. Based on our results, the best dose of ILE, as antidote for neurological manifestation of HA toxicity in rabbits, is 12 mL/kg, while Harvey and Cave recommended the 6 mL/kg of ILE as antidote for hemodynamic and cardiac toxicity of local anesthetic drugs [66]. This difference might be due to the variation of fat content of the original intoxicated tissues, brain and heart, which ILE should pull drugs out of. Moreover, Perez et al. has reported that the 18.6 mL/kg of ILE is the optimum dose as antidote for verapamil toxicity in rats [11]. This might also be due to animal variation.

## 5. Conclusion

In conclusion, ILE could reverse HA-induced neurotoxicity by lipid sink phenomena or direct effect of ILE. However, dose determination and safety of ILE in neurotoxicity need additional research.

## Figures and Tables

**Table 1:** Biochemical tests of haloperidol- (HA-) intoxicated rabbits receiving various doses of intravenous lipid emulsion (ILE) or normal saline, 24 hours after starting HA or AWD infusion. Data are reported in means ± standard error.

Lab test (unite)	ADW + normal saline 18 mL/kg	ADW + 18 mL/Kg ILE	HA + normal saline 18 mL/kg	HA + ILE 6 mL/kg	HA + ILE 12 mL/kg	HA + ILE 18 mL/kg
Blood urea nitrogen (mg/dL)	16 ± 1.68	18.0 ± 2.55	29.0 ± 8.45**	18.2 ± 3.11	23.4 ± 12.66	40.3 ± 18.52*
Serum creatinine (mg/dL)	1.16 ± 0.007	1.1 ± 0.063	1.82 ± 0.57	1.08 ± 0.08	1.14 ± 0.144	1.5 ± 0.141
Alanine transaminase (IU/L)	61.0 ± 5.1	62.5 ± 11.6	54.0 ± 13.4	57.3 ± 8.8	86.5 ± 4.6*	83.0 ± 8.5*
Aspartate transaminase (IU/L)	39.3 ± 6.3	67.8 ± 19.8	130.8 ± 63.5	42.5 ± 4.6	82.3 ± 29.8	314.0 ± 64.6*
Alkaline Phosphatase (IU/L)	243.5 ± 57.56	153.5 ± 38.86	379.5 ± 49.46	293.75 ± 23.83	342.2 ± 59.63	388.5 ± 48.5
Amylase (U/L)	274.4 ± 13.12	226.2 ± 29.05	234.6 ± 24.44	214 ± 19.20	269 ± 26.21	443.0 ± 120.9
Lipase (U/L)	215.5 ± 25.83	128.75 ± 33.62	147.0 ± 27.66	162.25 ± 42.13	226.5 ± 43.51	495.0 ± 228.5

*P < 0.05,**P < 0.01, ADW: aseptic distilled water.
